# Diazoxide protects against doxorubicin-induced cardiotoxicity in the rat

**DOI:** 10.1186/2050-6511-15-28

**Published:** 2014-05-27

**Authors:** Lisa Drange Hole, Terje Hjalmar Larsen, Kjell Ove Fossan, Fredrik Limé, Jan Schjøtt

**Affiliations:** 1 Section of Clinical Pharmacology, Laboratory of Clinical Biochemistry, Haukeland University Hospital, 5021 Bergen, Norway; 2 Institute of Biomedicine, University of Bergen, 5021 Bergen, Norway; 3 Department of Heart Disease, Haukeland University Hospital, 5021 Bergen, Norway; 4 Institute of Clinical Science, Faculty of Medicine and Dentistry, University of Bergen, 5021 Bergen, Norway

**Keywords:** Doxorubicin, Troponin T, Hydrogenperoxide, Doxorubicinol, Heart, Rat, 5-hydroxydecanoate, Cardiotoxicity, Ex vivo, Diazoxide

## Abstract

**Aim:**

Chemotherapy with doxorubicin is limited by cardiotoxicity. Free radical generation and mitochondrial dysfunction are thought to contribute to doxorubicin-induced cardiac failure. In this study we wanted to investigate if opening of mitochondrial K_ATP_-channels by diazoxide is protective against doxorubicin cardiotoxicity, and if 5-hydroxydecanoate (5-HD), a selective mitochondrial K_ATP_-channel antagonist, abolished any protection by this intervention.

**Methods:**

Wistar rats were divided into 7 groups (n = 6) and followed for 10 days with 5 intervention groups including the following treatments: (1) Diazoxide and doxorubicin, (2) diazoxide and 5-hydroxydecanoate (5-HD), (3) 5-HD and doxorubicin, (4) diazoxide and saline and (5) 5-HD and saline. On day 1, 3, 5 and 7 the animals received intraperitoneal (i.p.) injections with 10 mg/kg diazoxide and/or 40 mg/kg 5-HD, 30 minutes before i.p. injections with 3.0 mg/kg doxorubicin. One control group received only saline injections and the other control group received saline 30 minutes prior to 3.0 mg/kg doxorubicin. On day 10 the hearts were excised and Langendorff-perfused. Cardiac function was assessed by an intraventricular balloon and biochemical effects by release of hydrogen peroxide (H_2_O_2_) and troponin-T (TnT) in effluate from the isolated hearts, and by myocardial content of doxorubicin.

**Results:**

Doxorubicin treatment produced a significant loss in left ventricular developed pressure (LVDP) (p < 0.05) and an increase in both H_2_O_2_ and TnT release in effluate (p < 0.05). Diazoxide significantly attenuated the decrease in LVDP (p < 0.05) and abolished the increased release of H_2_O_2_ and TnT (p < 0.05). 5-HD abolished the effects of pretreatment with diazoxide, and these effects were not associated with reduced myocardial accumulation of doxorubicin.

**Conclusions:**

Pretreatment with diazoxide attenuates doxorubicin-induced cardiac dysfunction in the rat, measured by physiological indices and TnT and H_2_O_2_ in effluate from isolated hearts. The effect could be mediated by opening of mitochondrial K_ATP_-channels, reduced doxorubicin-associated free radical generation and decreased cardiomyocyte damage. Diazoxide represents a promising protective intervention against doxorubicin-induced acute cardiotoxicity.

## Background

Doxorubicin is a widely used chemotherapy drug, but its application is associated with cardiotoxicity. Free radical generation and mitochondrial dysfunction are thought to contribute to doxorubicin-induced cardiac failure [1,2]. Dose reduction protocols have been proposed to avoid the risk of delayed cardiotoxicity, but this might be at the expense of the anticancer effect [3]. In addition, impaired calcium handling and cellular damage mediated by reactive oxygen species, have been proposed as toxic mechanisms to explain both acute and delayed cardiotoxicity of anthracyclines [4-6].

Diazoxide has been in clinical use since the early 1960’s to treat severe non-malignant and malignant hypertension in hospitalized adults and acute severe hypertension in hospitalized children. Diazoxide is also used to treat hypoglycaemia. The mechanisms of diazoxide’s clinical action relate predominantly to the opening of pancreatic and smooth muscle K_ATP_-channels [7].

Mitochondria are the major effectors of cardioprotection by mechanisms that open the mitochondrial K_ATP_-channel, including ischemic and pharmacological preconditioning [8]. Pharmacological preconditioning, mimicking ischemic preconditioning, is suggested as an intervention to reduce doxorubicin cardiotoxicity [9]. Morphine has been demonstrated to mimic preconditioning in cardiomyocytes [10,11]. We have previously observed increased mortality in rats pretreated *in vivo* with morphine before doxorubicin (32). In a follow-up study we pretreated rats with morphine, isolated the hearts and exposed them to doxorubicin in a Langendorff perfusion system (unpublished data). We found that pretreatment with morphine *in vivo* was associated with a cardiodepressive effect in isolated hearts before doxorubicin exposure. After exposure to doxorubicin *ex vivo*, isolated hearts from rats pretreated with morphine were associated with increased release of hydrogen peroxide (H_2_O_2_), increased release of troponin-T (TnT), increased myocardial contracture and increased myocardial content of doxorubicin. These findings were in contrary to results from a comparable study [9] which found that morphine was protective against doxorubicin cardiotoxicity. Based on these opposing results we wanted to investigate further, whether pharmacological preconditioning could reduce doxorubicin cardiotoxicity.

Direct stimulation of myocardial δ_1_-opioid receptors leads to opening of mitochondrial K_ATP_-channels and a resultant increase in intracellular free radical signals *in vitro*[10]. Recently, nicorandil, a mitochondrial K_ATP_-channel opener, has successfully been administered to counteract the toxic effects of doxorubicin [12,13]. In light of our previous results, we now wanted to bypass the opioid receptors in this experiment, and study how direct opening of mitochondrial K_ATP_-channels interacts with the cardiotoxic mechanisms of anthracyclines. We used diazoxide, a selective mitochondrial K_ATP_-channel agonist, known to have protective properties against cardiac ischemia [7,14]. We also used 5-hydroxydecanoate (5-HD), a selective mitochondrial K_ATP_-channel antagonist. 5-HD inhibits the increase in free radicals seen with δ_1_-opioid receptor activation, and abolishes cardioprotection afforded by ischemic preconditioning [7]. Our model combines drug treatment *in vivo* with the study of hearts *ex vivo*, yielding direct physiological information on systolic and diastolic cardiac function combined with biochemical indices like H_2_O_2_, TnT and measurement of myocardial content of doxorubicin.

## Methods

### Materials

Doxorubicin was purchased from Meda AS (Slemmestad, Norway), diazoxide, 5-hydroxydecanoate and pentobarbital from Haukeland Hospital Pharmacy (Bergen, Norway), heparin from Leo Pharma A/S (Oslo, Norway), and ingredients for the Krebs-Henseleit bicarbonate buffer from Merck KGaA (Darmstadt, Germany). This study conforms to the Guide for the Care and Use of Laboratory Animals published by the US National Institutes of Health (NIH Publication No. 85–23, revised 1996) and was approved by the Animal Care and User Committee in Norway with document number: ID 1766 FOTS, and certified institution number 066 Vivarium, University of Bergen.

### Animals

Male Wistar rats weighing 200 ± 20 grams were purchased from Taconic (Ejby, Denmark). The animals were housed in grid-bottom metal wire cages in a room maintained at a 12 hour light/dark cycle at a temperature of 20–22°C. They were acclimatised for two weeks, housed three per cage and allowed free access to food pellets (Pellets rodent, Special Diets Services, Essex, UK) and tap water until i.p. injection of diazoxide, 5-HD, saline or doxorubicin. The animals were separated in individual cages based on their respective treatment protocols.

### Langendorff perfusion model

The perfusion medium was a modified, oxygenated (95% O_2_ and 5% CO_2_) Krebs-Henseleit bicarbonate buffer (KHBB) (pH 7.4) containing in mM: 118.5 NaCl, 25.0 NaHCO_3_, 1.2 MgSO_4_, 4.7 KCl, 1.2 KH_2_PO_4_, 11.0 D-glucose, and 1.25 CaCl_2_. Hearts were excised after anaesthesia of the rats with an i.p. injection of pentobarbital 50 mg/kg (0.1 ml/100 g bodyweight) and heparinised i.p. (0.1 ml 500 IU/100 g bodyweight). Anaesthesia was evaluated by the pedal-withdrawal reflex. The heart was rapidly excised and immediately placed in cold (4°C) KHBB to temporarily stop its beating and preserve it from ischemic injury prior to perfusion. The heart was mounted on a steel cannula placed in the aorta and perfused retrogradely in a Langendorff system with the use of thermostated (37°C) reservoirs (Lauda, Lauda-Königshofen, Germany), perfusion lines and heart chamber. Pressure regulated flow was performed at 100 cmH_2_O (73 mmHg), while volume regulated flow (12.5 ml/min) was performed by use of an Alitea peristaltic pump (Alitea, Stockholm, Sweden). A water-filled latex balloon was placed in the left ventricle and connected to a pressure transducer (Memscap AS, Skoppum, Norway) for the recording of left ventricular pressure (LVDP) and secondarily derived contractility indices. Left ventricular end-diastolic pressure (LVEDP) was adjusted between 4 and 8 mmHg. A second pressure transducer was connected to a side arm on the aortic cannula for the recording of aortic pressure (AoP), an index of coronary vascular resistance during volume-regulated perfusion. Pressure signals were amplified (Quadbridge, AD Instruments, London, UK) and recorded using a PowerLab data acquisition system (AD Instruments, East Sussex, UK). AoP, LVDP, LVEDP, left ventricular pressure first derivatives maximum (dp/dt_max_) and minimum (dp/dt_min_) were continuously displayed and recorded. Pacing (300 beats per minute by electric stimulation of 5 V amplitude of 3 ms duration) was obtained by placing one electrode on the right auricle and one on the steel cannula. Pacing was used to maintain a standard contractile response to the experimental drugs in the model not influenced by changes in heart rate and/or periods of arrhythmia. Coronary flow rate was measured by timed collection of the coronary perfusate that dripped from the heart. At the end of the perfusion protocol, hearts were removed from the Langendorff system and myocardial tissue from the left ventricle was dissected free and immediately frozen in liquid helium and stored at −80°C until analysis of doxorubicin and doxorubicinol was performed, within 14 days of termination of the perfusion protocol. Effluent samples of 1 mL were collected in 1.5 mL polypropylene Eppendorf micro test tubes (Eppendorf Vertrieb, Wesseling-Berzdorf, Germany) from each heart, at the end of the perfusion protocol, and stored at 0°C, until analysis for TnT within 4 days of termination of the perfusion protocol. Effluent samples of 1 mL were collected in Eppendorf tubes from each heart at the end of the perfusion protocol, placed in a thermostated (37°C) Eppendorf rack heated by a Lauda reservoir (Lauda, Königshofen, Germany), and immediately analysed for H_2_O_2_. All experiments and analysis were carried out between 7 am and 7 pm.

### Experimental design

**DIADOX** (n = 6) received pretreatment with an i.p. injection of 10 mg/kg diazoxide 30 minutes before an i.p. injection of 3 mg/kg doxorubicin.

**DIA5HDDOX** (n = 6) received pretreatment with an i.p. injection of 10 mg/kg diazoxide 30 minutes before an i.p. injection of 40 mg/kg 5-HD, 30 minutes before an i.p. injection of 3 mg/kg doxorubicin.

**5HDDOX** (n = 6) received pretreatment with an i.p. injection of 40 mg/kg 5-HD 30 minutes before an i.p. injection of 3 mg/kg doxorubicin.

**DIASAL** (n = 6) received pretreatment with an i.p. injection of 10 mg/kg diazoxide 30 minutes before an i.p. injection of 0.9% saline.

**5HDSAL** (n = 6) received pretreatment with an i.p. injection of 40 mg/kg 5-HD 30 minutes before an i.p. injection of 0.9% saline.

**SALSAL** (n = 6) received pretreatment with an i.p. injection of 0.9% saline 30 minutes before another i.p. injection of 0.9% saline.

**Figure 1:**
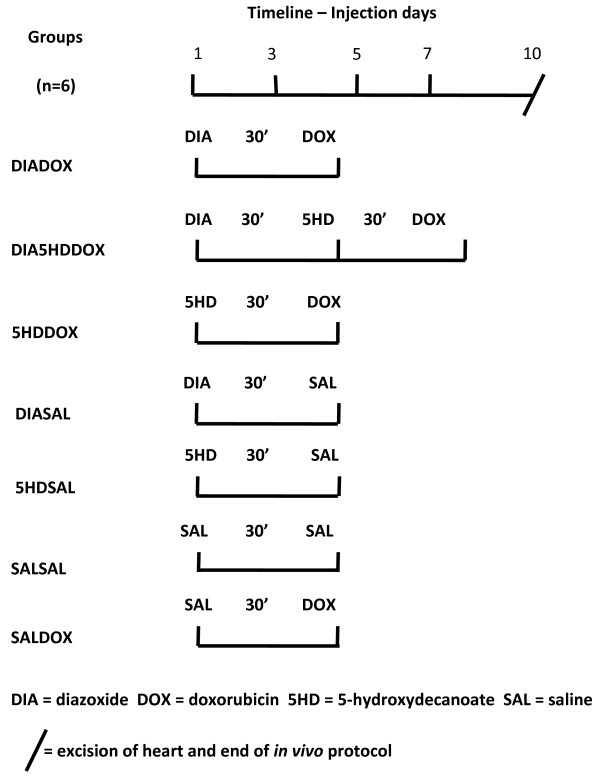
Drug treatment protocols.

**Figure 2:**
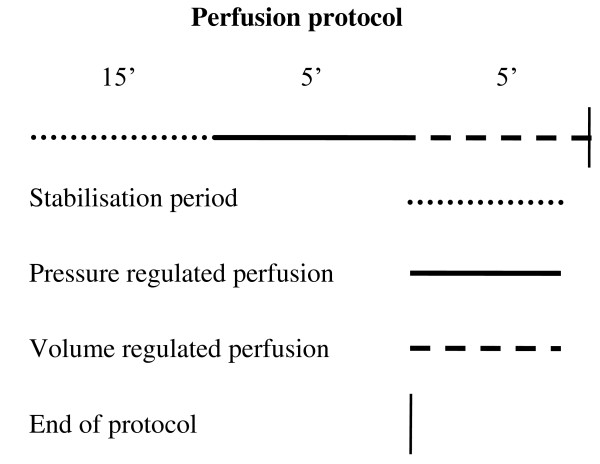
Perfusion protocol.

**SALDOX** (n = 6) received pretreatment with an i.p. injection of 0.9% saline 30 minutes before an i.p. injection of 3 mg/kg doxorubicin.Injections were given on day 1, 3, 5 and 7 and protocols are illustrated in Figure 1. On day 10 hearts were excised and Langedorff-perfused with the following protocol: 15 minutes stabilisation period with pressure-regulated flow, followed by 5 minutes with pressure-regulated flow and 5 minutes with volume-regulated flow. During the latter 10 minutes physiological data were recorded, and cardiac effluent samples collected for the evaluation of biochemical and pharmacological parameters. The perfusion protocol is illustrated in Figure 2.

### Quantification of doxorubicin and doxorubicinol

Doxorubicin and doxorubicinol were quantified by high performance liquid chromatography (HPLC-MS/MS) (1200 series RRLC, Agilent Technologies, USA) coupled to an Agilent 6410 triple quadrupole mass spectrometer using positive electrospray ionisation (Agilent Technologies, USA). Frozen left ventricular tissue was minced and weighted out in a glass tube with a screw cap and homogenized in physiological saline (2 ml/100 mg tissue) with a tissue homogenizer (Ultra Turrax, Sigma Aldrich, Germany). 1000 μl of sample was added 100 μl of daunorubicin as internal standard (IS), and 200 μl of buffer (1 M TRIZMA, pH 11.1) and mixed well before extraction with 4 ml ethylacetate/heptane (80/20 vol/vol). The samples were mixed using a rotary blender for 15 minutes and then centrifuged at 3500 rpm for 10 minutes at 10°C. The organic phase was evaporated to dryness at 50°C under nitrogen then dissolved in 100 μl of methanol followed by 100 μl of distilled water. The extract was mixed thoroughly and transferred to silanized vials before analysis. 25 μl of extract was injected and separated on a Zorbax SB-Aq (2.1 x 50 mm, 1.8 μm particles, Agilent Technologies, USA) column using gradient elution with acetonitrile and 0.1% formic acid in water. Quantification was performed using multiple reaction monitoring (MRM) mode at m/z 546.1 → 363.1 for doxorubicinol, m/z 544.1 → 361.1 for doxorubicin and m/z 528.1 → 321.1 for daunorubicin (IS).

### Effluent content of H_2_O_2_

H_2_O_2_ in cardiac effluent was measured using an Apollo 4000 electrochemical detection system (World Precision Instruments, Sarasota, Florida, USA). The electrode was calibrated using 9 serial dilutions of H_2_O_2_ in phosphate buffered saline with added aniline. The current recorded from the effluent was then calculated as μM H_2_O_2_. Samples were kept at 37°C during measurement. The electrode was allowed 3 minutes of stabilisation and 1 minute of recording.

### Effluent content of TnT

TnT in cardiac effluent was measured using an Elecsys 2010 immunoassay analyzer (Roche Diagnostics Norway AS, Oslo, Norway), based on the sandwich principle. Total duration of assay: 9 minutes. 1st incubation: 50 μL of sample, a biotinylated monoclonal cardiac TnT-specific antibody, and a monoclonal cardiac TnT-specific antibody labeled with a ruthenium complex (Tris(2,2-bipyridyl)ruthenium(II)-complex (Ru(bpy))) reacted to form a sandwich complex. 2nd incubation: After addition of streptavidin-coated microparticles, the complex became bound to the solid phase via interaction of biotin and streptavidin. The reaction mixture was aspirated into the measuring cell where the microparticles were magnetically captured onto the surface of the electrode. Unbound substances were then removed with ProCell. Application of a voltage to the electrode then induced chemiluminescent emission which was measured by a photomultiplier. Results were determined via a calibration curve which was instrument-specifically generated by 2-point calibration and a master curve (5-point calibration) provided via the reagent barcode. Detection limit was 5.0 ng/L.

### Statistics

All results are reported as mean values ± standard deviation (SD) in tables. Groups were compared with regards to parameters with a one-way analysis of variance (ANOVA) and subsequently Fisher’s protected least-significant difference test. SPSS for Windows version 17.0 was used and p < 0.05 was considered statistically significant.

## Results

All physiological results from the pressure regulated perfusion are presented in Table 1 and all physiological results from the volume regulated perfusion are presented in Table 2. Biochemical results and doxorubicin analysis are presented in Table 3.

**Table 1:** Physiological results from pressure regulated perfusion

	**DIADOX**	**DIA5HDDOX**	**5HDDOX**	**DIASAL**	**5HDSAL**	**SALSAL**	**SALDOX**
LVDP (mmHg)	136.6 ± 2.9*	122.9 ± 4.4	117.5 ± 5.2	146.4 ± 6.8*	144.6 ± 5.1*	152.7 ± 11.0*	120.4 ± 3.6
LVEDP (mmHg)	9.3 ± 1.5*	16.1 ± 1.8	14.9 ± 2.7	8.2 ± 1.5*	8.1 ± 1.6*	8.3. ± 1.6*	15.4 ± 2.2
AoP (mmHg)	73.0 ± 0	73.0 ± 0	73.0 ± 0	73.0 ± 0	73.0 ± 0	73.0 ± 0	73.0 ± 0
Heart rate (beats per minute)	300 ± 0	300 ± 0	300 ± 0	300 ± 0	300 ± 0	300 ± 0	300 ± 0
dp/dt_max_ (mmHg/s)	3525.5 ± 949.9	3295.3 ± 895.9	3283.4 ± 621.9	3647.4 ± 563.7	3522.5 ± 120.1	4499.1 ± 887.2	3026.6 ± 150.0
dp/dt_min_ (mmHg/s)	−2176.5 ± 1147.9	−2012.3 ± 282.3	−1952.7 ± 373.9	−2921.9 ± 200.3	−3050.9 ± 277.6	−3060.1 ± 311.3	−2173.0 ± 195.1
Coronary flow (ml/min)	11.3 ± 1.8*	9.5 ± 0.7	8.8 ± 1.2	11.8 ± 1.2*	12.0 ± 1.3*	12.6 ± 0.4*	8.9 ± 1.7

Values presented as mean ± standard deviation (SD). *Significantly different from hearts in SALDOX, p < 0.05.

**Table 2:** Physiological results from volume regulated perfusion

	**DIADOX**	**DIA5HDDOX**	**5HDDOX**	**DIASAL**	**5HDSAL**	**SALSAL**	**SALDOX**
LVDP (mmHg)	141.5 ± 6.9*	127.9 ± 4.3	108.1 ± 9.3	149.5 ± 10.8*	155.6 ± 13.0*	162.9 ± 14.7*	117.1 ± 9.1
LVEDP (mmHg)	9.6 ± 2.5*	16.0 ± 2.2	15.8 ± 2.1	10.6 ± 1.8*	8.2 ± 1.0*	9.3 ± 1.6*	18.0 ± 13.2
AoP (mmHg)	103.9 ± 2.2*	124.2 ± 5.1	130.5 ± 7.3	82.7 ± 6.0*	76.0 ± 3.5*	76.9 ± 3.6*	133.7 ± 5.0
Heart rate (beats per minute)	300 ± 0	300 ± 0	300 ± 0	300 ± 0	300 ± 0	300 ± 0	300 ± 0
dp/dt_max_ (mmHg/s)	3697.4 ± 141.0*	2512.9 ± 302.0	2950.7 ± 237.5	4583.6 ± 546.9*	3935.7 ± 213.6*	3973.6 ± 246.9*	2777.8 ± 386.9
dp/dt_min_ (mmHg/s)	−2380.6 ± 157.0	−2176.4 ± 154.8	−2530.1 ± 251.4	−2940.8 ± 261.6	−3041.6 ± 403.1	2862.0 ± 295.1	−2579.8 ± 329.1
Coronary flow (ml/min)	12.5 ± 0	12.5 ± 0	12.5 ± 0	12.5 ± 0	12.5 ± 0	12.5 ± 0	12.5 ± 0

Values presented as mean ± standard deviation (SD). *Significantly different from hearts in SALDOX, p < 0.05.

**Table 3:** Biochemical results and drug analysis

	**DIADOX**	**DIA5HDDOX**	**5HDDOX**	**DIASAL**	**5HDSAL**	**SALSAL**	**SALDOX**
Troponin -T effluate concentration ng/L	67.3 ± 7.9*	126.3 ± 20.5	130.0 ± 16.9	27.0 ± 4.9*	26.8 ± 4.9*	28.0 ± 5.3*	121.0 ± 17.2
H_2_O_2_ effluate concentration (μM)	54.9 ± 2.6*	72.8 ± 9.2	70.6 ± 5.7	24.8 ± 3.0*	26.1 ± 2.5*	22.8 ± 1.7*	73.5 ± 2.4
Doxorubicin tissue concentration nmol/g	1.9 ± 0.3	2.7 ± 0.4	3.3 ± 1.8	0 ± 0	0 ± 0	0 ± 0	2.6 ± 0.5
Doxorubicinol tissue concentration nmol/g	0.4 ± 0	0.7 ± 0.3	0.4 ± 0.1	0 ± 0	0 ± 0	0 ± 0	0.5 ± 0.2

Values presented as mean ± standard deviation (SD). *Significantly different from hearts in SALDOX, p < 0.05.

During pressure regulated perfusion LVDP was significantly (p < 0.05) higher in DIADOX (136.6 ± 2.9 mmHg) compared to SALDOX (120.4 ± 3.6 mmHg) and LVEDP was significantly (p < 0.05) lower in DIADOX (9.3 ± 1.5 mmHg) compared to SALDOX (15.4 ± 2.2 mmHg). There was no significant difference in LVDP, LVEDP or coronary flow in DIA5HDDOX or 5HDDOX versus SALDOX.

During volume regulated perfusion LVDP was significantly (p < 0.05) higher in DIADOX (141.5 ± 6.9 mmHg) compared to SALDOX (117.1 ± 9.1 mmHg). LVEDP was significantly (p < 0.05) lower in DIADOX (9.6 ± 2.5 mmHg) compared to SALDOX (18.0 ± 13.2 mmHg). AoP was significantly (p < 0.05) lower in DIADOX (103.9 ± 2.2 mmHg) compared to SALDOX (133.7 ± 5.0 mmHg). dp/dt_max_ was significantly (p < 0.05) higher in DIADOX (3697.4 ± 141.0 mmHg/s) compared to SALDOX (2777.8 ± 386.9 mmHg/s). dp/dt_min_ was not significantly different in DIADOX versus SALDOX, and there was no significant difference in LVDP, LVEDP, AoP, dp/dt_max_ or dp/dt_min_ in DIA5HDDOX or 5HDDOX versus SALDOX. There was no significant difference in dp/dt_max_ or dp/dt_min_ between any of the groups during pressure regulated perfusion.

Coronary flow was significantly higher (p < 0.05) in DIADOX (11.3 ± 1.8 ml/min) compared to SALDOX (8.9 ± 1.7 ml/min) during pressure regulated perfusion. There was no significant difference in coronary flow during pressure regulated perfusion in DIA5HDDOX or 5HDDOX versus SALDOX.

There was no significant difference in doxorubicin or doxorubicinol accumulation in myocard in any of the intervention groups versus SALDOX. TnT was significantly (p < 0.05) lower in DIADOX (67.3 ± 7.9 ng/L) compared to SALDOX (121.0 ± 17.2 ng/L). H_2_O_2_ concentration in effluate was significantly (p < 0.05) lower in DIADOX (54.9 ± 2.6 μM) compared to SALDOX (73.5 ± 2.4 μM). There was no significant difference in TnT or H_2_O_2_ in DIA5HDDOX or 5HDDOX versus SALDOX.

## Discussion

Pretreatment with diazoxide attenuated doxorubicin-induced cardiac dysfunction in the rat in the present study. Doxorubicin treatment produced a significant loss in left ventricular developed pressure (LVDP) (p < 0.05) both in volume- and pressure regulated perfusion similar to previous reports [15,16]. Diazoxide significantly attenuated this decrease in LVDP (p < 0.05) in both perfusion protocols. Diazoxide also improved diastolic dysfunction in doxorubicin treated hearts, by lowering the elevation of LVEDP (p < 0.05) in both perfusion protocols, compared to animals that received both diazoxide and 5-HD. The protective effect of diazoxide is equivalent to that of ischemic preconditioning, and diazoxide is often used as a pharmacological means to induce preconditioning [7]. The drug has previously been described as an agent with a unique molecular target by opening of mitochondrial K_ATP_-channels in cardioprotection. However, more recently a consensus seems to emerge that there are numerous effectors involved in the cardioprotective effects of diazoxide, and these effectors may synergistically contribute to its cardioprotective properties [7].

With volume regulated flow, the effects on doxorubicin-induced coronary vascular resistance, can be studied in parallel with effects on myocardial contractility. During volume regulated perfusion, aortic pressure, an indirect measure of coronary resistance, was increased in hearts that received both diazoxide and 5-HD, or just 5-HD, before doxorubicin. However, diazoxide pretreatment attenuated this effect. The vasodilatory effects of diazoxide in vascular smooth muscle are due to opening of vascular K_ATP_-channels. K_ATP_-channels have a pronounced role in controlling coronary blood flow and the coronary reserve, particularly in the resistance arterioles [17]. In some studies diazoxide has been noted to improve coronary flow, which is associated with cardioprotection, in perfused hearts [18], despite the fact that the coronary flow reserve is low in crystalloid-perfused hearts [19]. Coronary flow was significantly higher (p < 0.05) in DIADOX compared to SALDOX during pressure regulated perfusion. This effect was abolished with 5-HD. Based on these results, improved cardiac function associated with preserved coronary flow during pressure regulated perfusion, and attenuated increase in aortic pressure during volume regulated perfusion, could account for some of the cardioprotective effects of diazoxide.

Diazoxide pretreated hearts had lower concentrations of H_2_O_2_ in cardiac effluate compared to hearts that were pretreated with 5-HD and diazoxide or 5-HD alone. Diazoxide is an inhibitor of the mitochondrial complex II protein, succinate dehydrogenase (SDH). This inhibition also occurs in the heart [20]. The activity of SDH is a site of reactive oxygen species (ROS) generation, and the possibility has been raised that diazoxide and other SDH inhibitors mediate some of their cardioprotective effects via modulation of ROS production. Which ROS signals cardioprotection is mediated through is not fully understood [8]. Opening of mitochondrial K_ATP_-channels leads to increased ROS, leading to a persistent open state of the channel. The ROS responsible for this is not known [7]. H_2_O_2_ has been proposed as one candidate for this effect, although one study concludes that H_2_O_2_ is not the mediator of mitochondrial K_ATP_-channel-dependent ROS signalling [8]. In our study we found higher levels of H_2_O_2_ in effluate from hearts that had not undergone diazoxide pretreatment before doxorubicin, or that had received both 5-HD and diazoxide before doxorubicin. The level of H_2_O_2_ associated with cellular signalling is much lower than the levels associated with cardiotoxicity [21]. The present study was not designed to examine signalling effects, and our observations are thus associated with the reduced cardiotoxicity of doxorubicin. This is supported by our observation of diazoxide improving diastolic dysfunction in doxorubicin treated hearts, by lowering the elevation of LVEDP. Diastolic dysfunction and contracture is proposed to be related to ROS generation [22,23].

Diazoxide pretreated hearts also had lower concentrations of TnT in cardiac effluate compared to hearts that were pretreated with 5-HD and diazoxide or 5-HD alone. Troponins are myocardial regulatory proteins, which regulate the calcium mediated actin and myosin interaction. Troponin-T is widely used as a specific marker to diagnose myocardial infarction. Doxorubicin is associated with increased TnT in serum and in heart effluate [15,24,25].

Finally, pretreatment with diazoxide was not associated with decreased myocardial accumulation of doxorubicin or doxorubicinol which suggest that the protective effects did not involve a change of distribution of the anthracycline to the heart *in vivo*.

### Limitations

In this study we wanted to investigate if opening of mitochondrial K_ATP_-channels by diazoxide is protective against doxorubicin cardiotoxicity. Mitochondrial K_ATP_-channels have been implicated as important mediators of preconditioning [26,27]. However, the evidence for their role in preconditioning is primarily based on the effects of pharmacological agents, in particular, diazoxide and 5-HD. Diazoxide has been reported to be a specific opener of mitochondrial K_ATP_ channels, but studies have suggested that its mechanism of cardioprotection may be due to other actions, such as inhibition of succinate dehydrogenase [28,29], activation of sarcolemmal K_ATP_ channels [30] or transient opening of the mitochondrial permeability transition pore [31]. In addition, it might be argued that the complex metabolic effects of 5-HD severely limit its usefulness as a ‘selective’ blocker of mitochondrial K_ATP_ channels [32]. Furthermore, since their identification heavily relies on the use of diazoxide as a specific opener and 5-HD as a specific blocker, the very existence of mitochondrial K_ATP_ channels may be questioned. This skeptical view is supported by a study in which no changes in mitochondrial matrix volume induced by diazoxide or 5-HD could be detected [33]. Hence, one view on 5-HD is that it should no longer be considered a useful tool for studying the role of mitochondrial K_ATP_ channels in preconditioning [32]. Thus, although diazoxide attenuated doxorubicin-induced cardiac dysfunction in our results, our model was not designed to establish the precise protective mechanisms. Furthermore, we cannot exclude that the protective effects involved a change in distribution of doxorubicin to different compartments within the heart.

## Conclusion

The main observation in the present results is that pretreatment with diazoxide attenuates doxorubicin-induced cardiac contractile dysfunction, and attenuates release of biomarkers of cardiotoxicity in effluate without decreasing the accumulation of doxorubicin. 5-HD completely abolished this effect of diazoxide in our rat model. A possible mechanism could be opening of mitochondrial K_ATP_-channels. However, we cannot exclude other effects of the drug, including opening of vascular K_ATP_-channels. The low specificity of 5-HD, calls for additional studies of mechanisms of a promising protective intervention.

## Abbreviations

5-HD: 5-hydroxydecanoate; H_2_O_2_: Hydrogen peroxide; TnT: Troponin-T; LVDP: Left ventricular developed pressure; KHBB: Krebs-Henseleit bicarbonate buffer; LVEDP: Left ventricular end-diastolic pressure; AoP: Aortic pressure; dp/dt_max_: Left ventricular pressure first derivatives maximum; dp/dt_min_: Left ventricular pressure first derivatives minimum; HPLC-MS/MS: High performance liquid chromatography; MRM: Multiple reaction monitoring; SD: Standard deviation; ANOVA: Analysis of variance; ROS: Reactive oxygen species; SDH: Succinate dehydrogenase.

## Competing interest

The authors declare that they have no competing interests.

## Authors’ contributions

LDH and KOF carried out the pharmacological studies, and drafted the manuscript. LDH carried out the immunochemical analysis and the animal experiments. LDH and FL carried out the hydrogen peroxide analysis, and drafted the manuscript. LDH, THL and JS conceived of the study, participated in its design and coordination, performed the statistical analysis, and helped to draft the manuscript. All authors read and approved the final manuscript.

## Pre-publication history

The pre-publication history for this paper can be accessed here:

http://www.biomedcentral.com/2050-6511/15/28/prepub
